# Invited Discussion on: Lipo-Bodylift Reconstruction Following Massive Weight Loss—Our Experience with 100 Consecutive Cases

**DOI:** 10.1007/s00266-021-02133-6

**Published:** 2021-03-15

**Authors:** Dennis J. Hurwitz, Armando A. Davila

**Affiliations:** 1 Hurwitz center for Plastic Surgery, Plastic Surgery University of Pittsburgh, 3109 Forbes Avenue, Pittsburgh, Pa I5213 USA; 2 Hurwitz center for Plastic Surgery, UNLV School of Medicine, 3109 Forbes Avenue, Pittsburgh, Pa I5213 USA

*Level of Evidence V* This journal requires that authors assign a level of evidence to each article. For a full description of these Evidence-Based Medicine Ratings, please refer to Table of Contents or online Instructions to Authors www.springer.com/00266.

In the preceding article, “Lipo-Bodylift reconstruction following massive weight loss: Our experience with 100 consecutive cases” [1], the distinguished innovative plastic surgeon scholars at the Rennes University Hospital analyse their recent experience with a circumferential lower body lift that removes subcutaneous fat through preliminary liposuction and then excises the skin. They achieved their stated goals of… “reconstruction of a skin envelop that considered the patient’s body mass index”, … an acceptably reconstructed bodily image…and reduced complications”.

They seek to demonstrate comparable safety profiles with the technique in patients with predominantly vertical tissue laxity with and without moderate adiposity. The patients were prospectively documented by demographics, medical history, BMIs, bariatric procedure, operative data, complications and revisions. While appreciating their premise that connective tissue pathology after MWL was critical to the design of their procedure, these discussants struggle to practically apply the four-level classification since measuring magnitude and orientation of tissue laxity is both inexact and variable across the body. Moreover, the classification system does not meaningfully correlate with degree of weight loss, which can be a stronger predictor of laxity then BMI and subjective examination alone. Indeed, only their Level IV acknowledges transverse skin laxity, which we feel needs to be addressed in all candidates for body lifts, particularly those with an average of 50 kg of weight loss, the average in this study.

They summarize their previously published technique [2] of radical excision site liposuction with preservation of connective tissue, excision of only skin and then multilayered interrupted suture closure of neighbouring flaps after limited to no undermining. Little to no liposuction of the epigastric flap was done and undermining was only along a narrow path from the umbilicus to the xyphoid. Their 27 patients with delayed wound healing was a distinct improvement over their initial 40% rate. Excluded were patients with significant lipodystrophy (Class III) or transverse skin laxity (Class IV), prior non-weight loss abdominal surgery and with obvious wide Diastasis Recti. Arguably, Class III candidates potentially benefit from more extensive liposuction due to more excessive adiposity and of greater concern for surgeons regarding safety. The Class II patients ultimately have minimal liposuction outside of the resection area performed, which speaks to their already reasonable body habitus with median liposuction volumes low at 2,800 cc.’s, as was the mean mass of resected skin at 904.5 g. The median operative time, apparently for only Lipo-Bodylift, was a respectable 153.5 min, with a steep learning curve starting from roughly 220 min, which by itself explains reduced complications. Physiotherapy was initiated after 6 weeks, which we feel should be started at four days.

The Frenchmen compare their wound healing improvement and advantage in the lower body to the well-described closure of intact medial thigh and arm flaps over performed excision site liposuction. Their prior researched established that the tissue remaining after liposuction exhibits a morphologically preserved microvascular network, suggestive of functional preservation [3]. We concur that preservation of draining lymphatic and veins of the medial upper arms and thighs clearly reduces lymphoceles and prolonged distal swelling. However, similar extensive vascular drainage is not present in the low back, which does not suffer from prolonged oedema or lymphocele. It is more likely that the minimal dissection performed is more preservative of the vascular network, but it is unclear whether in Class II patients the liposuction is necessary to achieve that result.

Missing from “Lipo-Bodylift” is a survey of patient satisfaction or analysis of aesthetics. The only relevant comment is the need for seven secondary liposuctions for under correction. They show four cases with before and sometime thereafter frontal, posterior and lateral views. Critical comparison is difficult because arms are in various positions and upper body clothing partially obscure. No comment is made regarding deformity or changed aesthetics. These authors have previously pronounced that, “the benefit to patients is mainly functional, not esthetic” [4], but they do not surgically address the functional benefit of rectus plication with an argument that its functional benefit has been proved to be minimal. Nevertheless, they agree that parietal laxity may hamper the aesthetic results of the waist due to persistent laxity in the abdominal wall. Ultimately, while the discussants applaud the creativity and persistence to lower complications, we feel that improving aesthetics is an essential goal of lower body lift surgery. While removal of unwanted hanging tissue pleases patients, creating a tight skinned feminine shape should be our goal. In over 100 cases of oblique flankplasty with lipoabdominoplasty (OFLA) [5], the senior discussant has usually achieved that aesthetic along the full range of MWL deformity with less than 20% rate of complications.

**Fig. 1:**
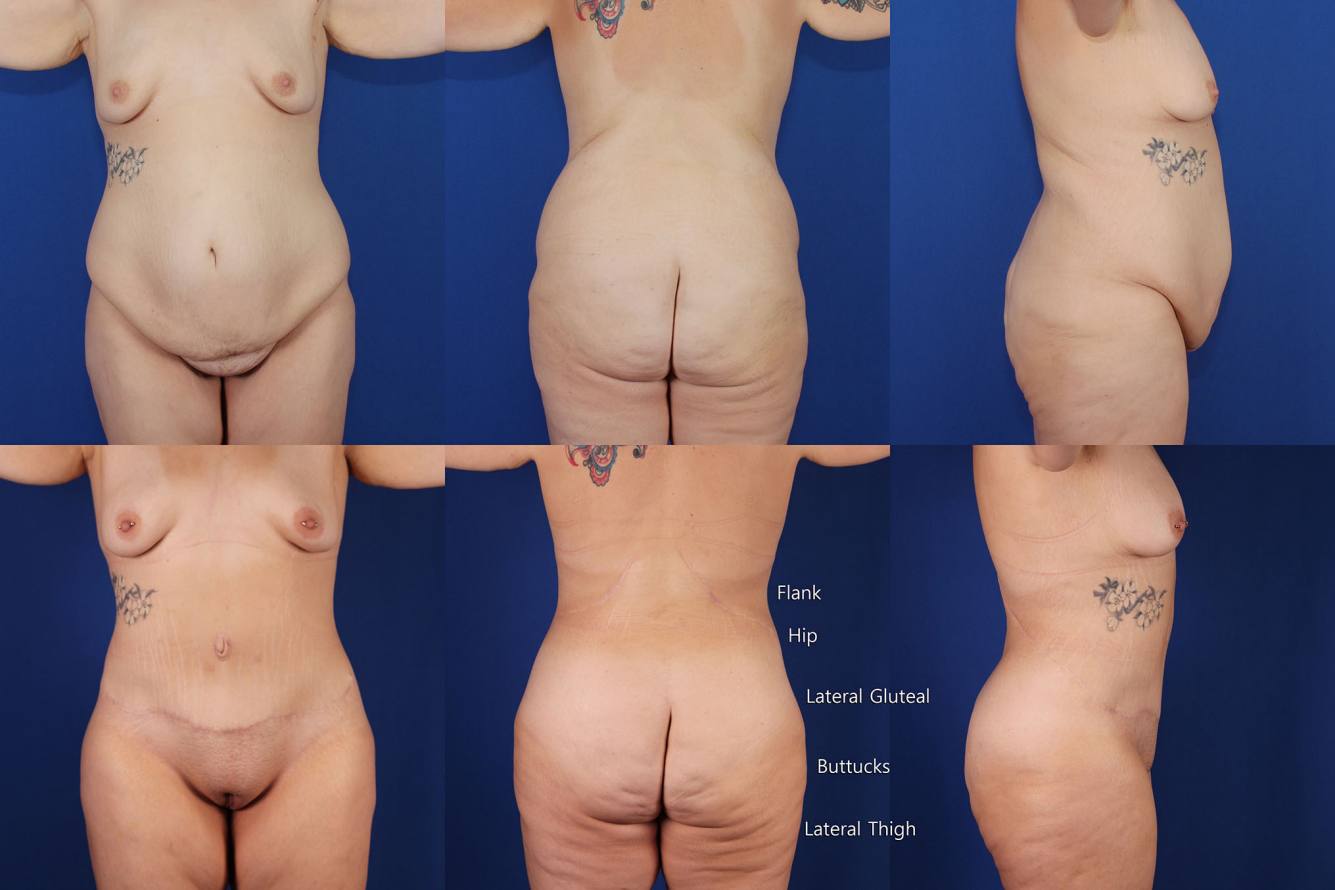
A selected case from senior discussant with deformity shown in upper row like Fig. 2 in Lipo-Bodylift. Lower row are same views 8 months following oblique flankplasty with lipoabdominoplasty (OFLA) and lipoaugmentation of the breasts, which should be compared to views in lower row Fig. 2 Lipo-Bodylift. The post-operative lower posterior view is labelled with right flank, hip, lateral gluteal, buttock and lateral thigh, which were preoperatively obscured by hanging tissues and are now distinct and attractively seen

**Fig. 2:**
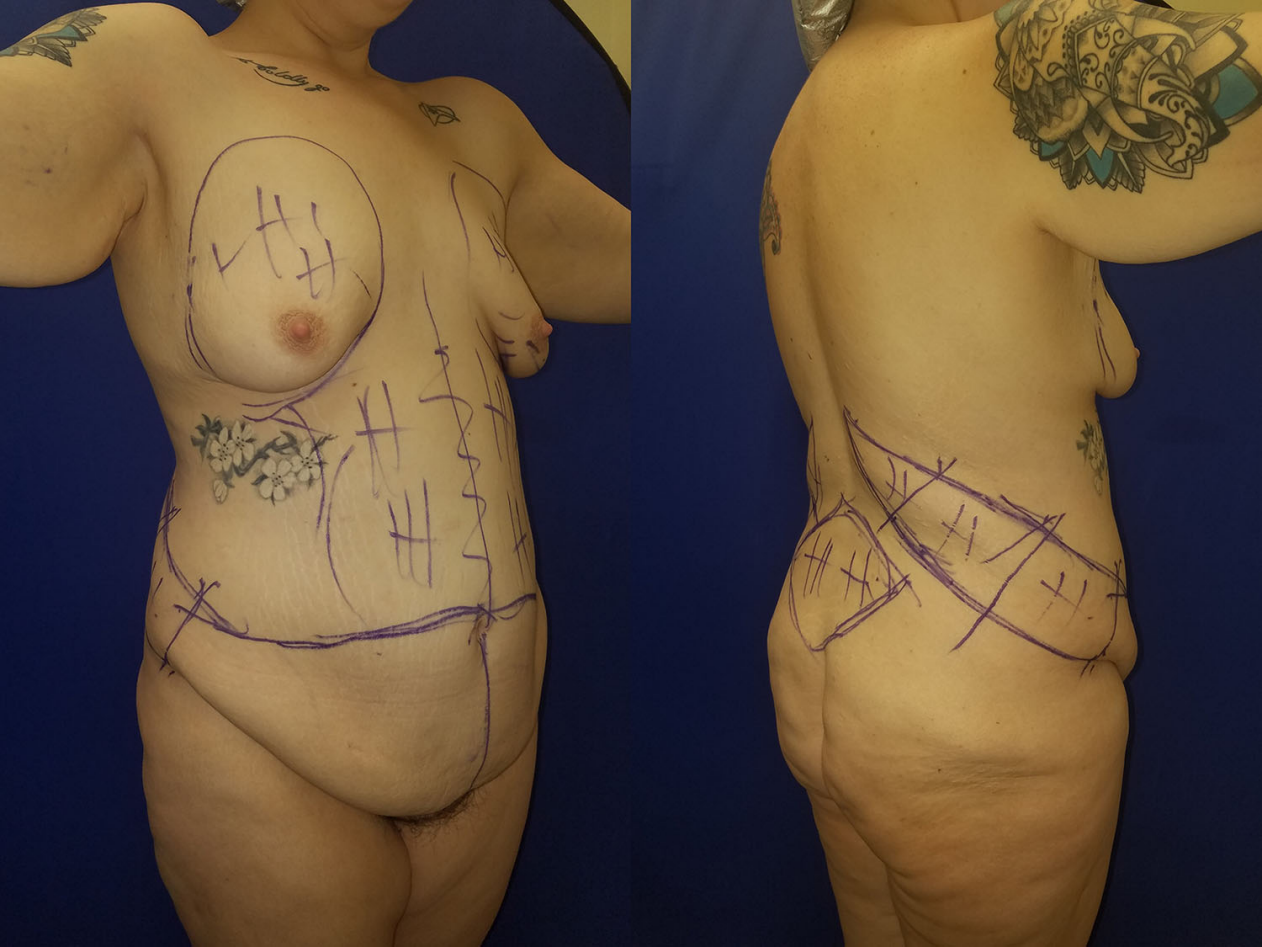
Immediate preoperative right oblique views of surgical markings for OFLA and VASERlipo of flankplasty excision site and epigastrium and 160 cc. lipoaugmentation of breast

To support the assertion that pleasing aesthetics can be achieved, we have selected a single OFLA case with deformity similar that seen in Lipo-Bodylift (Fig. 2) type II [1]. Figure 1, upper row, is of a 25-year-old, where two years after gastric bypass her BMI went from 55 to a stable 28.6. Both cases have multiple roles of abdominal fatty protuberance with the lower roll hanging over sagging mons pubis and lateral hips, indicative of both vertical and horizontal skin laxity. The flank and hip bulging complex fill the posterior waist. Lateral gluteal depressions lie inferior to broad hip prominences. The lateral buttocks have sagged into the lateral thighs. Their midtorso folds are more clearly shown in Fig. 1.

Before assessing the post-operative aesthetics of Fig. 1 lower row, examin Fig. 2 which shows the right-side preoperative markings for the OFLA and minimal VASERlipo of 300 cc, the flank excision site allows for non-undermined closure and from epigastrium 160 cc outside the site of excision to further contour the abdomen. Centred over the bulging flanks, the right oblique flankplasty seamlessly continues into the broad lower abdominal excision. Through the medial vertical component of the oblique excision and the posterior rotation advancement of the superior flap, transverse excess is further reduced, thereby narrowing and tightening the waist.

The bottom row of Fig. 1 is 8 months after a complication-free OFLA with only the suprapubic scar obvious. While no plication of the rectus muscle was done, the abdomen is flat with a curvaceous narrow waist and slight lower abdominal convexity. The flanks are broadly narrow with smooth transition from the lower costal margin to the hips. Figure 2 in “Lipo-Bodylift” shows residual abdominal skin rolls with no narrowing of the waist. Superior to a constricted circumferential scar are distorted muffin top, full flanks and persistent midback folds. The lateral thighs are the widest (saddlebags), unlike the lateral buttocks in Fig. 1. Whether examined analytically or viewed as a whole construct, the tight skinned feminine result of Fig. 1 is near ideal.

While our oblique low back scar location runs counter to low excision dogma of traditional body lift surgery, the incredibly improved contours, tight skin, faded scars and low rate of complications, despite VASERlipo both anteriorly and posteriorly, demonstrate superb aesthetics while adhering to the safety concepts outlined in this study. Moreover, OFLA with VASERlipo can apply to the Class I–IV candidates in this study with slight modifications. While this impressive presentation of Lipo-Bodylift appears to be narrow in scope, we accept the authors conclusions that thoughtful, limited and targeted liposuction can improve outcomes with minimal adverse sequalae. As their contribution is not sufficient to define female form, we opine that surgeons expand their horizons to attain function and improve aesthetics, and compare your own outcomes to Lipo-Bodylift and OFLA for your thoughtful conclusion.
